# 

**Published:** 1852-02

**Authors:** 


### NOTICE TO CORRESPONDENTS.

We decline, with regret, the very interesting history of the *Northern Dispensary*, with which we have been favored—as hardly coming within the province of a Journal of Medicine.

The Case of *Constipation* we should be much pleased to insert; but it offers no point of novelty, although evincing much practical tact and sound judgment on the part of the author.

The case by *Medicus* will be published, if authenticated by the narrator's name.

Communications and Books for notice should be addressed to the Editors, care of Messrs. Lindsay & Blakiston.

Letters, &c., connected with the *business affairs* of the Journal should be addressed to the Publishers.

Papers for publication must be received *before* the 20th of the month, or they cannot appear in the forthcoming number.

The following Journals have been received in exchange:

New York Medical Times.
Medical News. Philadelphia.
American Journal of Medical Sciences.
New Jersey Medical and Surgical Reporter.
New York Medical Gazette.
The Boston Medical and Surgical Journal. (Weekly.)
Stethoscope and Virginia Medical Gazette.
Western Journal.
Buffalo Journal.
Transylvania Medical Journal.
Nashville Journal of Medicine and Surgery.
Southern Medical and Surgical Journal.
Western Lancet.
Northern Lancet.
New Hampshire Journal of Medicine.
New Orleans Monthly Medical Register.
Opal, from the Utica Insane Asylum.
Dental Times and Advertiser.
The London Lancet. (Weekly, London.)
The Medical Times. (Weekly, London.)
Dublin Medical Press.
Journal of Pharmacy.
North Western Medical Intelligencer.
British-American Journal of Medical and Physical Science.
American Journal of Insanity. Oct.

---

### BOOKS RECEIVED.

Hassall's Microscopic Anatomy, by Vanarsdale. American Edition.
Alin on Retro-pharyngeal Abscess.
Transactions of American Medical Association. Vol. IV.
Operative Surgery of Bernard and Huette, translated by Drs. Van Buren and Isaacs, New York.
Cazenave on the Skin, by Backley. New edition.
Hooker on Homœopathy.
Dickson on Sleep, Pain, Life and Death.
Meigs' Memoir of Dr. S. G. Morton.

---

Communications have been received from:—
Dr. T. H. Yardley, of Philadelphia.
Dr. H. Y. Smith, Philadelphia.
Dr. S. Weir Mitchell, Philadelphia.

---

The foreign correspondents of the Examiner will please direct their Exchanges and other communications to the care of Mr. Thos. Delf, No. 12 Paternoster Row, London, or Mr. H. Bossange, 21 Bis, Quai Voltaire, Paris.

